# Pyogenic brain abscess, a 15 year survey

**DOI:** 10.1186/1471-2334-12-332

**Published:** 2012-11-30

**Authors:** Jannik Helweg-Larsen, Arnar Astradsson, Humeira Richhall, Jesper Erdal, Alex Laursen, Jannick Brennum

**Affiliations:** 1 Department of Infectious Diseases, Copenhagen University Hospital, Rigshospitalet, Denmark; 2 Department of Neurosurgery, The Neuroscience Centre, Copenhagen University Hospital, Rigshospitalet, Denmark; 3 Department of Neurology, Herlev Hospital, Copenhagen, Denmark; 4 Department of Infectious Medicine, Aarhus University Hospital, Skejby, Denmark

**Keywords:** Brain abscess

## Abstract

**Background:**

Brain abscess is a potentially fatal disease. This study assesses clinical aspects of brain abscess in a large hospital cohort.

**Methods:**

Retrospective review of adult patients with pyogenic brain abscess at Rigshospitalet University Hospital, Denmark between 1994 and 2009. Prognostic factors associated with Glasgow Outcome Score (GOS) (death, severe disability or vegetative state) were assessed by logistic regression.

**Results:**

102 patients were included. On admission, only 20% of patients had a triad of fever, headache and nausea, 39% had no fever, 26% had normal CRP and 49% had no leucocytosis. Median delay from symptom onset to antibiotic treatment was 7 days (range 0–97 days). Source of infection was contiguous in 36%, haematogenous in 28%, surgical or traumatic in 9% and unknown in 27% of cases. Abscess location did not accurately predict the portal of entry. 67% were treated by burr hole aspiration, 20% by craniotomy and 13% by antibiotics alone. Median duration of antibiotic treatment was 62 days. No cases of recurrent abscess were observed. At discharge 23% had GOS ≤3. The 1-, 3- and 12-month mortality was 11%, 17% and 19%. Adverse outcome was associated with a low GCS at admission, presence of comorbidities and intraventricular rupture of abscess.

**Conclusions:**

The clinical signs of brain abscess are unspecific, many patients presented without clear signs of infection and diagnosis and treatment were often delayed. Decreased GCS, presence of comorbidities and intraventricular rupture of brain abscess were associated with poor outcome. Brain abscess remains associated with considerable morbidity and mortality.

## Background

Historically, brain abscesses were usually caused by contiguous infection following sinusitis or middle-ear infection and carried a poor prognosis [1,2], but as a result of improved neuro-imaging, surgery and antibiotic treatment, brain abscesses are now relatively rare, and mortality has improved.

Several aspects of management remain controversial, including the need for surgery and optimal surgical approaches, type and length of antibiotic treatment, and need for monitoring during treatment. No randomized controlled prospective trials are available.

We describe brain abscesses diagnosed during a 15-year period. We report microbial and treatment characteristics in relation to outcome for patients with pyogenic brain abscesses treated with a combination of surgery and antibiotics or antibiotics alone. A particular focus is placed on a detailed analysis of diagnostic delays as well as type and length of antimicrobial treatment in relation to outcome.

## Methods

We retrospectively reviewed adult patients ≥ 16 years of age, diagnosed with confirmed or suspected pyogenic brain abscess at the Departments of Neurosurgery, Infectious Medicine and Neurology at Rigshospitalet University Hospital, Copenhagen, between January 1994 and April 2009. Cases were ascertained through ICD 10 discharge codes. Rigshospitalet is a tertiary, regional and national specialist referral hospital. During the study period, we estimate that approximately 1.7 million people at risk of brain abscess were primarily served by Rigshospitalet. Medical records were reviewed by paper and electronic data query. Data were entered by custom made data entry forms in Epidata and cross-validated [3]. Contiguous infection was defined as infection secondary to otitis media, mastoiditis, sinusitis or dental infections. One patient was lost to long-term follow-up by emigration, 6 months after hospital discharge. Complete follow-up for the remaining patients was available to January 2011. Inclusion criteria were characteristic computed tomography (CT)/magnetic resonance imaging (MRI) findings and evidence of bacterial brain abscess from surgery, autopsy and/or appropriate microbiological specimens (Cerebrospinal (CSF), blood, sputum) or characteristic CT/MRI findings, a clinical history and treatment response compatible with pyogenic brain abscess in patients with negative microbial findings. Patients with mycobacterial, parasitic or fungal abscesses and cases with subdural empyema were excluded.

### Antibiotic treatment

The choice of antibiotic treatment was primarily guided by microbial findings; the majority of patients were treated with 4–8 weeks of i.v antibiotics, often followed by a period of peroral antibiotic treatment. Overall, the first-choice for treatment was a beta-lactam (high dose penicillin, cephalosporin or carbapenem) in combination with metronidazole or other drugs for anaerobic coverage. The usual treatments were high-dose penicillin or either cefuroxime or ceftriaxone in combination with metronidazole for abscess secondary to ear, nose and throat (ENT) infection and in more recent years meropenem in combination with metronidazole for culture-negative infections. The choice and length of antibiotic treatment were not uniform; changes in treatments were common, based on individual microbial findings, tolerability and/or the treating physicians’ preferences. Over time, an increasing number of patients were treated with meropenem, both as empiric treatment before culture results and after definitive microbial diagnosis.

### Statistical analysis

Data were analysed using Stata, version 11.2 (StataCorp). Pearsons chi-square and Fischer’s exact test was used as appropriate. The primary outcome measures were 3-month mortality after admission date and Glasgow Outcome Score (GOS) at discharge. Logistic regression was used to examine outcome. In these analyses, odds ratios (OR) together with robust 95% confidence intervals (CI) are presented. A p-value of less than 0.05 (two-sided) was considered significant.

Permission to access patient records was obtained in accordance with the Danish Act on processing of personal data and the study was approved by the Copenhagen ethics committee.

## Results

A total of 102 patients were diagnosed with brain abscess between January 1994 and April 2009. Median age was 47 years and 65% were male. This yields an estimated incidence of 0,4/100.000/year. Table 1 provides baseline characteristics.

**Table 1:** Characteristics at admission

**Characteristics**	**Number of patients (%)**
Male	65 (64%)
Age, median (range)	47 (16–81)
Comorbidities *	47 (46%)
Diabetes	6 (6%)
Alcohol	4 (4%)
Malignancy	6 (6%)
Renal failure	4 (4%)
Surgery	8 (8%)
IVDU	7 (7%)
HIV	2 (2%)
Congenital heart disease/endocarditis	4 (4%)
Other	11 (11%)
**Presenting symptoms and signs**	
Fever	61 (60%)
Neck stiffness	26 (25%)- missing data on 10% of patients
Headache	73 (72%)
Nausea/ vomiting	41 (40%)
Focal neurological deficits	58 (57%)
Seizures	21 (21%)
Impaired consciousness	46 (45%)
Fever and headache and nausea/vomiting	20 (20%)
Fever and headache and focal deficits	23 (23%)
GCS at presentation	
12-15	79 (77%)
8-11	10 (10%)
<8	9(9%)
Missing GCS- score	4 (5%)

*: 12 patients had more than one comorbidity. GCS: Glasgow Coma Score, IVDU: Intravenous Drug User.

### Clinical findings

Relatively few patients (20%) presented with the classical triad of fever, headache and nausea (Table 1). The most common presenting symptom was headache (72%), followed by documented fever on admission or a history of fever (60%), however only 22% had documented temperature elevation in excess of 38.5°C at presentation. Fifty-eight (57%) patients presented with focal neurological deficits, including paresis, aphasia, visual deficits, ataxia, and cranial nerve paresis. Glasgow Coma Score (GCS) was less than 15 at admittance in 54% of the patients.

### Laboratory findings

Complete laboratory recordings on admission were available for approximately 80% of the patients, of which two thirds had elevated CRP but only one half had leucocytosis -Table 2.

**Table 2:** Laboratory findings

**Laboratory findings**	**Number of patients (%)**
CRP, mg/l	
0-20	28 (27%)
20-100	22 (22%)
>100	33 (32%)
Missing admission CRP	19 (19%)
Leukocytosis (>11.000 cells/μl)	49 (48%)
Missing leucocyte count	15 (15%)
Lumbar puncture, n	36 (35%)
CSF cell leucocytes	
0-15	7 (19%)
15-100	4 (11%)
100-1000	10 (28%)
1000-10.000	15 (42%)

Lumbar puncture was performed in one third of the patients, typically before the final diagnosis of brain abscess was made. Of these patients, 19% had no pleocytosis, 11% low grade pleocytosis (< 100 cells/microliter) and 70% cerebrospinal fluid (CSF) leukocytosis (> 100 cells/microliter).

### Neuroradiological investigations

The primary neuroimaging study was CT in 88% and MRI in 12% of cases. During diagnostic workup or monitoring of treatment, MRI was used (in addition to or instead of CT) in 35% of patients. The most common abscess locations were the frontal (37%) and parietal lobes (27%)-Table 3. In 21% of cases, multiple (>1) abscesses were observed. The majority of frontal abscesses were caused by contiguous spread, in contrast to parietal and multiple abscesses which were more commonly seen with haematogenous spread. However, as shown, the cerebral location of abscess did not accurately correlate with the source of infection.

**Table 3:** Portal of entry in relation to microbial findings, location and source of brain abscess

	**Portal of entry**				
	**Contiguous**	**Haematogenous**	**Postneurosurgical/trauma**	**Unknown**	**Total**
	**N=37**	**N=29**	**N=9**	**N=27**	**102**
**Microbe**					
Streptococcus species	19	16	1	19	55
Staphylococci	4	5	5	2	16
Gram-negative bacteria	5	1	1	1	8
Anaerobic bacteria	6	3	2	3	14
Nocardia	1	1	0	0	2
Other	1	1	0	2	4
Negative culture	5	3	1	5	14
**Abscess Location**					
Frontal	15	8	6	8	37
Parietal	5	11	3	8	27
Temporal	8	4	0	2	14
Occipital	3	3	0	3	9
Pontine	0	3	0	0	3
Cerebellar	5	0	0	3	8
Basal ganglia	1	1	0	2	4
Multiple abscesses	3	11	3	4	21
**Source of abscess**					
Sinusitis	13				13
Dental	15				15
Otogenic	9				9
Lung		9			9
Endocarditis/congenital heart disease		4			4
Abdominal/urinary infection		4			4
Other		12			11
Postneurosurgical/trauma			9		9
Unknown				27	27

### Comorbidities and predisposing factors

Co-morbidities were present in 46% of patients (Table 1). Eight patients had had previous surgery, of which two cases were secondary to surgery for brain tumours. Only six patients were immunocompromised, two from HIV and four due to long-term steroid therapy and/or chemotherapy. In two patients with nocardial brain abscess, one had Waldenstrom’s macroglobulinemia and one had received prolonged high dose steroids for chronic obstructive pulmonary disease (COPD).

### Diagnostic delay

Median delay from symptom onset to first admission and to surgery was 3 days (range 0–82 days) and 10 days (range 0–98 days), respectively. Median delay from symptom onset to initiation of antibiotic treatment was 7 days (range 0–97 days).

The majority of patients (81%) were initially admitted to peripheral hospitals, before referral. Median delay from admission to operation for patients transferred from other hospitals was 5 days (range 0–99 days) compared to 4 days (range 0–28) for patients directly admitted to non-neurosurgical departments at Rigshospitalet. In 8 patients referred from other hospitals, brain tumours were primarily suspected at the time of referral based on symptoms, signs and interpretation of initial neuro-imaging, which all were CT. Additionally, two patients were primarily suspected of stroke, based on CT findings.

### Microbial findings

By surgery, microbiological material was obtained from 89 of the 102 patients**-** Table 3 and 4. By direct microscopy of surgically obtained gram-stained pus, bacteria were seen in 64 (72%) patients, of which 23 (26%) cases had findings compatible with mixed species infection. Cultures from pus were positive for bacteria in a total of 82 (92%) cases, in 72 as single species and in 11 cases as mixed growth. In 7 (8%) patients, pus was culture negative, among these cases; bacteria were detected by microscopy in 4 cases. In the remaining patients, organisms were most often detected in blood or CSF. CSF cultures were positive in 6 of 36 (17%).

**Table 4:** Bacterial pathogens

**Bacterial pathogens**	**No. of isolates**	**%**
**Streptococcus species**	**55**	**54%**
Streptococcus milleri group	32	
Streptococcus oralis/sanguis	3	
Group A streptococcus	1	
Streptococcus pneumoniae	4	
Streptococcus bovis	1	
Streptococcus species	14	
**Staphylococci**	**16**	**15%**
Staphylococcus aureus	14	
Coagulase-negative staphylococci	2	
**Gram-negative bacteria**	**8**	**8%**
E.coli	2	
Haemophilus spp.	5	
Pseudomonas	1	
**Anaerobic bacteria**	**17**	**17%**
Fusobacteria	4	
Actinomyces	2	
Prevotella	2	
Peptostreptococci	2	
Other anerobic bacteria	7	
**Nocardia**	**2**	**2%**
**Other species**	**4**	**4%**
Negative culture, n= patients	14	14%
Mixed bacterial pathogens	11	11%
Primary microscopy compatible with several different bacterial species	23	23%
Positive gram-stain from pus/CSF	64	64%
Gram-stain positive/negative culture	4/65	
Negative gram-stain and positive culture	18	
Negative culture from brain pus	7	

The most common species were streptococci, particularly from the *S.milleri* group, followed by anaerobic bacteria, staphylococci and gram-negative bacteria. There were two cases of cerebral nocardiosis and two cases of actinomycosis. Six patients had nosocomial brain abscess.

Among the 7 patients with negative cultures from surgery, 4 patients (75%) had received antibiotics before surgery for a median duration of 13 days. Of the 82 patients with positive cultures, 32 (39%) had received antibiotics before surgery, for a median duration of 4.5 days- these differences were not significant. Four of 10 patients (40%) with mixed infections compared to 32 of 79 (41%) without mixed infection had received pre-surgical antibiotics, p=0.98.

### Treatments

The majority of patients, 89%, were treated surgically**-** Table 5. Burr hole aspiration was the preferred primary operation for abscess evacuation and also when repeat evacuation was necessary. In a minority of cases, however, individual surgeon preferences may have dictated the use of a craniotomy instead of burr hole aspiration. Cases where craniotomy was the primary surgical evacuation procedure, included those cases where a neoplasm was initially suspected, warranting a craniotomy, or in the case of postoperative abscess following a craniotomy for intracranial neoplasm or other lesions.

**Table 5:** Treatments

**Treatments**			**Range**
Median antibiotic treatment, overall		62 days	0-261 days
Median time of intravenous treatment		41 days	0-111 days
Median antibiotic time postsurgery		59 days	1-245 days
No. of patients receiving antibiotics before surgery		48 (54%)	
No. of patients receiving oral antibiotics after intravenous		36	
Duration of po treatment after iv, median		44 days, range 10-405	
Management			
Surgery		89 (87%)	
	Aspiration	68 (67%)	
	Craniotomi	21 (20%)	
Conservative, medical		13 (13%)	
Antibiotic treatments			
Penicillin		68%	
Cephalosporin		63%	
Carbapenem		43%	
Metronidazole		83%	
Quinolone		21%	
Fucidic acid		39%	
Rifampin		20%	
Intrathecal Antibiotics (Vancomycin)		10 (10%)	

Of patients treated surgically, 76% were managed by burr hole or stereotactic aspiration and 24% by craniotomy. The 13 non-surgically treated patients had either inaccessible or multiple abscesses or had a primary presentation of meningitis. Three of these patients died early after admission, two immediately, and one 11 day after admission.

In 33 (33%) patients, the primary surgical intervention was followed by repeated aspiration, craniotomy or the insertion of external ventricular drainage. There were relatively few perioperative complications (n=7), most often haemorrhages and all non fatal. All patients were initially treated with intravenous antibiotics, except one patient who died few hours after admission. Changes of intravenous antibiotic treatment were common and 37% of patients were treated with three or more different antibiotic regimens. The majority of patients were treated with a combination of a beta-lactam agent and metronidazole. During antibiotic treatment, 68% received high-dose penicillin, 63% a cephalosporin and 43% a carbapenem). For additional anaerobic coverage, 83% received metronidazole and 22% clindamycin. In addition, fucidic acid (n=40), rifampicin (n=21), quinolones (n=18), cotrimoxazole (n=2), chloramphenicol (n=3) and linezolid (n=3) were used. Intrathecal vancomycin was administered to 10% (n=10) of patients in addition to intravenous therapy, of which seven had intraventricular rupture of brain abscess.

### Treatment in culture negative patients

The primary beta-lactam treatment among the 14 patients with negative cultures was a cephalosporin in 7 patients, a cephalosporin followed by penicillin in one case, penicillin in two cases and cephalosporin followed by meropenem in two cases; all patients also received metronidazole. In addition, one patient was treated with chloramphenicol and one patient died immediately after admission. No clear association between empiric choice of treatment and outcome was found, six of the 14 patients died within three month, of which three patients had been treated with cephalosporins and one patient treated with a cephalosporin followed by penicillin.

### Duration of antibiotic treatment

The median duration of antibiotic treatment was 62 days. Among patients treated surgically, postoperative intravenous treatment was continued for a median of 41 days (range 1–111). Intravenous treatment was followed by oral antibiotics in 36 (36%) of patients, for a median duration of 44 days (range 10–245 days).

There was no difference in the duration of intravenous or total length of antibiotics between patients treated by either burr hole aspiration compared with craniotomy or between patients treated surgically compared with antibiotics only (p=0.34). To compare patients with primary craniotomy without need of reoperation with patients managed by primary aspiration and subsequent need for reoperations, the length of antibiotic treatments was analyzed; Although the length of postsurgical intravenous antibiotic was somewhat reduced among the 17 patients managed by initial craniotomy without repeat surgical interventions compared to the 29 patients in which primary aspiration were followed by repeat aspiration/operations (median 40 days versus 49 days, p=0.21), the total duration of antibiotic treatment (intravenous and peroral) was comparable (median 76 days versus 70 days, p= 0.35).

In 28 (31%) of patients managed by surgery, the total duration of postsurgical antibiotics was less than 6 weeks, and in 61 (69%) more than 6 weeks. In eight of the 28 patients, treatment was terminated because of death. Among the remaining 20 patients, 2 died, one due to the progression of a glioblastoma, and one primarily due to liver failure. No relapse of abscesses was observed in the remaining 18 patients.

There was a trend towards extended antibiotic therapy in patients monitored by MRI compared with only CT; Among surviving patients, 69% of the patients monitored by MRI were treated for more than 60 days compared with 50% of patients monitored by CT (p=0.08).

### Steroid treatment

Before surgery, 29% were instituted on steroid treatment. After surgery, 52% received steroids, usually for a limited time period.

### Outcome

The 1- month, 3-month and 1-year mortality rates were 11%, 17% and 19%, respectively. At discharge, a GOS ≤ 3 (death, severe disability or vegetative state) was observed in 23% of patients. No cases of recurrent abscesses were observed.

**Figure 1:**
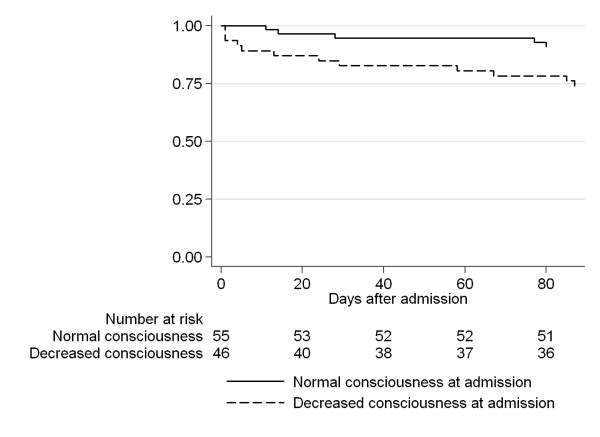
Kaplan-Meier analysis of survival in relation to decreased consciousness at admission.

Predictors of adverse outcome (GOS ≤ 3) are shown in Table 6. In univariate logistic regression analysis, GCS at admission, age, the presence of comorbidities, meningitis (defined as positive spinal fluid and/or CSF leukocytosis) and intraventricular rupture of brain abscess were associated with adverse outcome. However, in multivariate analysis only GCS at admission, the presence of co-morbidities and intraventricular rupture of brain abscess were significant predictors of outcome. Additional predictors previously reported to be associated with a poor outcome, namely fever (>38.5°C), diagnostic delay and advanced age were not associated with adverse outcome in our material [4-6]. The presence of leucocytosis was associated with adverse outcome (GOS≤ 3) in 29% of patients compared to 11% without (p=0.039), however, as the outcome was equivalently poor (33% with GOS≤ 3) in the 15 patients with missing values for leukocytes, this predictor was excluded from analysis. Mortality according to consciousness at admission is shown in Figure 1.

**Table 6:** Predictors of adverse outcome, logistic regression analysis

**Characteristic**	**Good outcome**	**Adverse outcome**	**Univariate**	**Multivariate, OR, 95% CI, p-value**
	**GOS > 3, alive**	**GOS≤3/death**	**OR, 95% CI**	
Age, mean (SD)	45 (15.9)	52 (15.6)	1.03, 1.0-1.06	1.02,0.98-1.07, p=0.28
No predisposing factors	48	7 (13%)	3.5, 1.3-9.4	9.3, 1.6-54.7, p=0.014
Comorbidity	31	16 (34%)		
GCS at presentation				
12-15	68	11(14%)	1.0	1.0
8-11	7	3 (30%)	1.7, 0.6-12	3.5, 0.7-17.5, p=0.113
<8	1	8 (89%)	26.5, 3.6-381	80.6, 2.5-2574, p=0.013
Burr hole aspiration	54 (79%)	14 (21%)	1.0	
Craniotomy	17 (81%)	4 (19%)	0.9, 0.3-3.1	
Medical	8 (62%)	5 (38%)	2.4, 0.7-8.5	
Duration of symptoms before admission, days median (IQR)	4.0 (0–10)	3.0 (0–7)	0.99, 0.97-1.03	
Time from admission to surgery, days median (IQR)	5.0 (1–13)	7.0 (1–14)	1.02, 0.97-1.07	
Intraventricular rupture of brain abscess	4 (40%)	6 (60%)	6.6 ,1.7-26.2	11.5, 2.5-53.2, p=0.002
Meningitis*	20 (25%)	11 (48%)	2.7, 1.03-7.1	1.5, 0.3-6.3, p=0.597
Streptococcus species	45 (82%)	10 (18%)		
Staphylococci	9 (60%)	6 (40%)		
Gram-negative species	5 (83%)	1 (17%)		
Anaerobic bacteria	7 (87%)	1 (13%)		
Other	4 (100%)	0		
Negative culture	9 (54%)	5 (36%)		
Total n	79 (77%)	23 (23%)		

GOS: Glasgow Outcome score, GCS: Glasgow Coma Score, IQR: Inter quartile range. Bacterial species were omitted from logistic regression analysis due to limited number in each group and no significant difference in outcome in univariate analysis. *: Defined as positive spinal fluid culture and/or elevated CSF leukocytes (>15 cells/ μl).

There was no difference in outcome (GOS≤ 3, 36% vs. 20%, p=0.2) or need for reoperation (14% vs 35%, p=0.12) when patients with unknown microbial aetiology were compared with those with known bacterial infection.

The re-operation rate was significantly higher in patients managed by burr hole aspiration; 43% underwent a repeat procedure compared to 19% with craniotomy, p=0.05, 95% CI: There was, however, no significant difference between patients managed by burr hole aspiration compared to craniotomy, in the duration of antibiotics or in outcome according to GOS at discharge. Intraventricular rupture of brain abscesses was observed in 10 patients. This was associated with a poor outcome (GOS≤ 3) in 6 of 10 patients at discharge.

Overall, the outcome of patients receiving antibiotics before surgery compared to patients who first were initiated on treatment at surgery appeared inferior (GOS≤ 3, 33% vs. 11%, p=0.011). However, since the decision to administer antibiotics before operation and outcome was critically influenced by presence of severe infection at admission, which would be reflected in an elevated CRP, outcome was reanalyzed according to CRP at admission. The outcome of patients with elevated admission CRP (> 20 mg/l) was inferior to patients presenting with a normal CRP (GOS ≤3 29% vs 7%, p=0.025). No association between outcome and administration of pre-surgical antibiotics was found when adjusted for CRP at admission.

Steroid treatment before surgery did not affect outcome, as 21% versus 23% had GOS ≤ 3 at discharge. Similarly, the outcome for patients treated post-surgically with steroids was comparable to non-treated patients (GOS≤ 3: 25% vs. 20%).

## Discussion

Despite diagnostic advancements with the introduction of CT and later MRI, as well as better antimicrobial agents and neurosurgical procedures, brain abscess continues to be a serious, potentially life threatening condition. Mortality continues to be relatively high. In our study almost a quarter of patients had a poor outcome.

The key prognostic factors were level of consciousness at admission and presence of comorbidity. These findings confirm previous observations. Mortality has generally been reported to be higher with severe mental state changes [4,7], and rapidly progressing neurological impairment [8,9] and with predisposing illness [9,10]. Intraventricular rupture was associated with a poor prognosis; of 10 patients, 3 died and 3 had severe neurological deficits at discharge. This observation confirms previous findings by Takeshita and co-workers [11,12]. In particular, increased meningeal irritation and/or localized enhancement of the ventricular wall adjacent to the abscess are predictors of subsequent rupture and need to be recognized and managed particularly aggressively [11,12].

Our study has several strengths. In particular, the complete availability of detailed diagnoses, neuroimaging, microbiological and follow-up data provided the opportunity for detailed analysis of the characteristics and management of brain abscess in relation to outcome. We believe all patients diagnosed with pyogenic brain abscess at our institution were identified, including both surgically and non-surgically treated patients as well as patients who died very early after admission.

A similar Danish study by Nielsen et al. of 200 patients, diagnosed with cerebral abscess at Rigshospitalet between 1935 and 1976 including patients managed before the availability of penicillin and CT scans, showed a decrease in mortality from 78% before 1945 to 37% between 1945 and 1958 and to 17% between 1958 and 1976 [13]. Of note, this mortality rate is similar to the 17% 3-months mortality rate observed in our study, suggesting that the overall outcome has changed little in recent years. However, as patients with brain abscess are heterogeneous, the outcome is critically dependent on baseline characteristics. In agreement with other studies from the last three decades we observed an increased rate of patients with unknown source of infection (25%) and/or concurrent morbidity (46%), compared to the previous study at by Nielsen in which only 15% had unknown source of infection and 25% concurrent morbidity [13].

Recent retrospective studies from East Asia and Europe have reported marked variations in the outcome of brain abscess with mortality rates ranging from 6% to 35% and adverse outcome ranging from 10 to 58% [4-10,14-25]. The highest mortality rates have been reported in case-series with a high proportion of non-surgically managed patients [9,15], primarily reflecting the overall poor prognosis of patients with severe septic shock, meningitis, multiple abscesses and/or staphylococcal infections. In contrast, case series with a high proportion of surgically managed streptococcal abscesses tend to have a much better overall outcome [6,14,19,23].

A substantial number of patients (n=36) were investigated by lumbar puncture. The CSF findings varied considerably with CSF cellularity ranging from normal to high, emphasizing the need for early adequate neuroimaging in patients suspected of meningitis and low level (< 1000 cells/μl) pleocytosis, as 39% had CSF leucocytes between 15 and 1000 and 19% normal values. In agreement with previous studies, the microbial yield of lumbal puncture was low, with only 16% being culture positive. Furthermore, lumbar puncture is generally contraindicated in patients with suspected intracranial mass lesions, such as an abscess due to risk of herniation. Blood cultures were also rarely positive, but should yet always be considered, as they are minimally invasive and may yield the pathogens in some instances [4,26].

Diagnostic delay has generally been believed to be a key contributing factor to the severity and outcome of brain abscess [6,27]. In our study, the majority of patients presented with uncharacteristic symptoms and diagnosis was frequently delayed. The usual signs of infection such as fever, increased WBC and increased phase reactants were only present in 60% at presentation. Although fever was the single most common presenting symptom, 42% displayed no febrile symptoms. It is a common misconception that fever is necessarily a part of the presenting scheme of cerebral abscess. In several series fever has been reported to occur in about 50% or less of cases, and its absence should never be used to exclude the diagnosis of brain abscesses [5,10,16,18,27,28].

The significant delays observed in timely diagnosis and treatment were caused by both “doctors delay” (delay or misinterpretation of neuro-imaging, referral and definitive surgery) and “technical delays” (caused by inadequate initial choice of imaging, e.g. CT without contrast). For example, in 10 patients, the initial CT imaging study was interpreted as being compatible with brain tumour or stroke. Importantly, access to urgent MRI was not always readily available. The differentiation between abscess and brain tumours is not always straightforward. A typical clue on MRI is the presence of a hypointense capsule on T2-weighted images, however a capsule may be absent and while advanced techniques such as Magnetic Resonance Spectroscopy (MRS) or brain- Positron Emission Tomography(PET)–CT, may improve the differential diagnosis between brain abscess and brain tumours, no single technique achieves perfect specificity and sensitivity, and a definitive diagnosis of brain abscess may not be reached until surgery [29-31].

In agreement with other studies, contiguous, haematogenous and unknown primary source of infection constituted approximately a third of cases, respectively [18,19]. While several textbooks have suggested that the location of the abscess may predict the source of the infection, we found that abscess location had an overall limited predictive value for the primary source of infection. For instance, we observed that cerebellar abscesses were often not associated with otic infection. Similar observations have been reported by Kowleesar et al. [14].

Despite a relatively heterogeneous spectrum of bacterial species in this study, the predominant species remained streptococci. In agreement with Xiao [9], the duration of antibiotic treatment before operation was not directly related to the culture positive rate. Of note, 15 patients with positive surgical cultures had received antibiotics for more than 5 days prior to surgery, implying that surgical intervention is often essential to attain full therapeutic response. Compared to other studies, the presence of anaerobes was relatively infrequent (17% of cases).

For several years in Denmark, high dose penicillin in combination with metronidazole was recommended as first-line treatment for most cases of brain abscess, since penicillin resistant streptococci and MRSA are uncommon in Denmark. In recent years, broader-spectrum agents have been increasingly used based on the good outcome of third line cephalosporins or carbapenems in small non-controlled studies and the increased rate of patients with culture negative disease or unknown source of infections [19,20].

In our study, the efficacy of individual antibiotic choice is difficult to evaluate as changes of antibiotics were common during treatment. Recent data based on 16sRNA PCR suggest that brain abscesses contain a high number of fastidious and/or unculturable species [32]. However, we found little indication that broad spectrum treatment with cephalosporin or meropenem was superior to standard high-dose penicillin combined with metronidazole therapy in patients managed by surgery. Hence, the extent to which more detailed microbial identification could contribute to improved treatment is unknown.

Although the use of steroid treatment in brain abscess is controversial, corticosteroids may be beneficial in patients with raised intracranial pressure and potentially life-threatening complications such as impending cerebral herniation [26,33]. Our policy is to reserve corticosteroid treatment to patients with significant cerebral edema with mass effect, compromising mental or neurological status, despite maximal surgical treatment. Almost a third of patients received steroids before surgery, including a few patients initially suspected of brain tumours and more than half of surgically treated patients received at least temporary treatment with steroids after surgery. We were unable to demonstrate any clear benefit or risk associated with steroid use in agreement with previous studies [7,8,34].

The appropriate duration of antibiotic treatment has been debated [35,36]. In the absence of solid evidence, most recommendations are vague and based on minimum duration of therapy. In a recent British study, inadequate duration (< 3 weeks) or choice of oral antibiotic therapy, after switch from intravenous therapy was reported to be associated with recurrence of abscess among eight patients [21]. Nevertheless, treatment duration as short as two to three weeks after definitive surgical intervention has been used in selected patients with success [35,37]. American textbook recommendations [38] are primarily based on a review paper by Mathisen [33], which suggests high-dose intravenous agents for 6 to 8 weeks followed by oral antimicrobial therapy for 2 to 3 months if an appropriate therapy is available. UK guidelines recommend a minimum of 4–6 weeks of therapy if the abscess has been excised or aspirated and 6–8 weeks if treated conservatively [36].

Most patients had a prolonged duration of antibiotic treatment. While we observed no cases of recurrence, the rationale for extended duration of therapy may be questioned. Among 18 patients, for which the duration of postsurgical antibiotic treatment was limited to less than 6 weeks, we observed no cases of recurrence. In our study, treatment duration was usually guided by regression of abscess as verified by CT or MRI and all patients had at least one or more follow-up neuroimaging studies. Paradoxically, while the use of MRI was often critical for a timely diagnosis, subsequent MRI monitoring was frequently associated with prolongation of treatment. Even with effective treatment, MRI radiologic changes such as the disappearance of contrast enhancement lag behind clinical improvement and enhancement may persist for months. In several cases, the continued presence of such signs prompted clinicians to extend the duration of therapy despite clinical improvement.

The majority (89%) of patients were treated surgically. The patients who were treated conservatively with antibiotics in our series often had multiple abscesses, poorly accessible abscesses, such as in the cerebellum, or a poor premorbid conditions. Seventy-six% of patients requiring surgical treatment had needle aspiration performed, while 24% of those requiring surgical treatment underwent a craniotomy with excision of the abscess. These proportions are similar to those reported in other series [17,27], but are still a matter of debate and highly dependent on surgeon preference. It is likely that in our series, an even higher proportion of needle aspirations would have been sufficient, had the preoperative diagnosis of brain abscess been less equivocal, as a differential diagnosis of intracerebral tumour, warranting a full craniotomy, was initially suspected in several cases preoperatively. Additionally, in the rare cases that abscess developed as a complication to a prior craniotomy, was a craniotomy usually performed for abscess excision. Several studies suggest that needle aspiration is as effective as abscess excision in the management of the majority of intracerebral abscesses and excision can be reserved for abscesses that fail to regress despite aspiration, or that are caused by resistant pathogens [33]. In a recent retrospective literature review, the mean mortality appeared considerably lower in patients managed by aspiration compared to surgical excision, but did not provide information on measures such as need for reoperation or recurrence [23]. In our series, the need for reoperation was not surprisingly higher in patients managed by aspiration compared to excision, however, the duration of antibiotic treatment was similar.

## Conclusion

In conclusion, despite a decline in mortality rates in the era of advanced neuroradiological studies and better antibiotics, brain abscess continue to be a serious condition with high mortality risk. The clinical signs of brain abscess are often unspecific and diagnosis is often delayed, particularly in patients initially investigated by CT compared to MRI. Key determinants of poor outcome are decreased GCS at admission, the presence of comorbidities and intraventricular rupture of brain abscess.

## Competing interests

The authors declare that they have no financial or non-financial competing interests.

## Authors’ contributions

JH-L, AA, AL and JB planned the study. JH-L, AA, HR, JE and AL undertook the data extraction. JH-L was responsible for data analysis, the initial manuscript draft, correction of the article and submission. All authors contributed to the discussions and interpretation of data, and rviewed the final report.

## Pre-publication history

The pre-publication history for this paper can be accessed here:

http://www.biomedcentral.com/1471-2334/12/332/prepub
